# Why are essential genes essential? - The essentiality of *Saccharomyces* genes

**DOI:** 10.15698/mic2015.08.218

**Published:** 2015-07-25

**Authors:** Zhaojie Zhang, Qun Ren

**Affiliations:** 1 Department of Zoology and Physiology, University of Wyoming, Laramie, WY 82071, USA.

**Keywords:** cell death, cell survival, essential genes, growth conditions, Saccharomyces cerevisiae

## Abstract

Essential genes are defined as required for the survival of an organism or a cell. They are of particular interests, not only for their essential biological functions, but also in practical applications, such as identifying effective drug targets to pathogenic bacteria and fungi. The budding yeast *Saccharomyces cerevisiae* has approximately 6,000 open reading frames, 15 to 20% of which are deemed as essential. Some of the essential genes, however, appear to perform non-essential functions, such as aging and cell death, while many of the non-essential genes play critical roles in cell survival. In this paper, we reviewed and analyzed the levels of essentiality of the *Saccharomyces cerevisiae* genes and have grouped the genes into four categories: (1) Conditional essential: essential only under certain circumstances or growth conditions; (2) Essential: required for survival under optimal growth conditions; (3) Redundant essential: synthetic lethal due to redundant pathways or gene duplication; and (4) Absolute essential: the minimal genes required for maintaining a cellular life under a stress-free environment. The essential and non-essential functions of the essential genes were further analyzed.

## DIFFERENT LEVELS OF ESSENTIALITY

**Figure 1:**
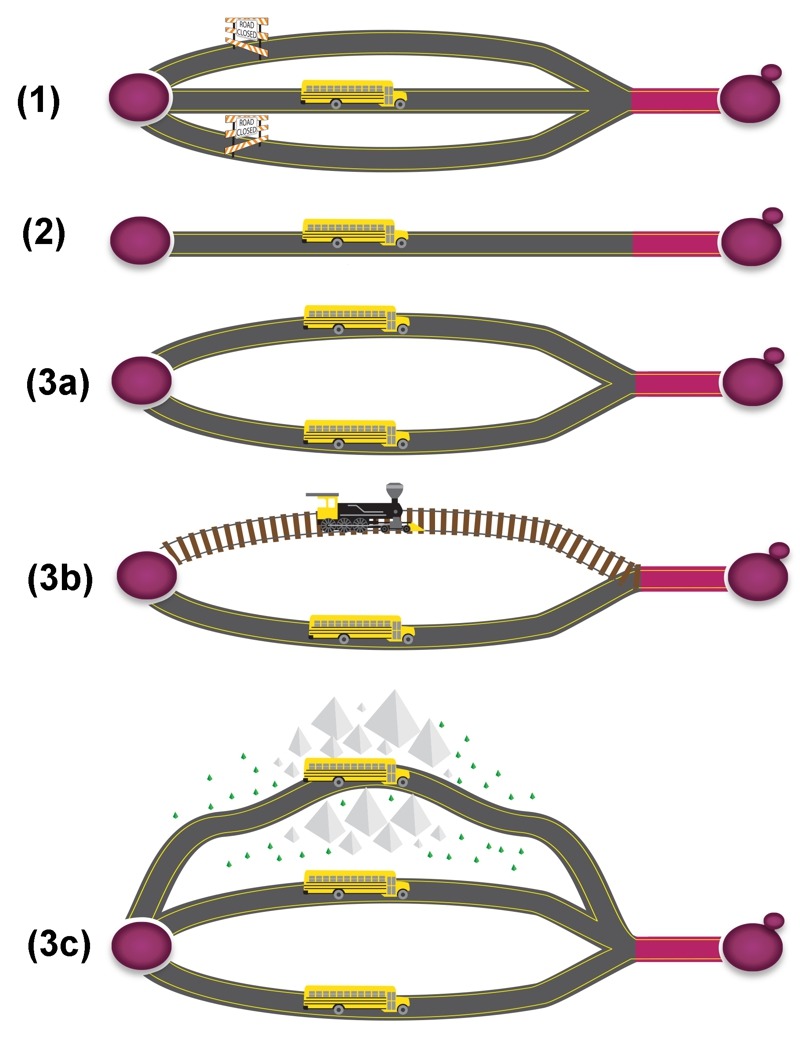
FIGURE 1: A symbolic illustration of different levels of essentiality of the *Saccharomyces cerevisiae* genes. **(1)** Conditional essential: when all other roads are closed, this alternative road becomes essential. **(2)** Essential: the only available road to your destination. **(3a)** Redundant essential: two duplicated/similar roads to your destination; **(3b)** Redundant essential: two different roads (pathways), but to the same destination; **(3c)** Redundant essential (synthetic sick): Two similar/different roads, plus a less preferred road, when the main roads become unavailable. The last part of the road (marked as red) represents the absolute essential.

Generally speaking, essential genes are genes required for a cell or an organism to survive. Disruption or deletion of such genes causes cell death, indicating that these genes perform essential biological functions. Some of the essential genes, however, appear to have non-essential functions. *FAS2*, for example, encodes an α-subunit of fatty acid synthetase, which catalyzes the synthesis of long-chain saturated fatty acids 1. The null mutant could be rescued by fatty acid supplement. It is not clear why *FAS2* functions as an essential gene. This also makes the term essential somewhat confusing, because essentiality depends on the environment in which cells live 2. For example, a yeast gene required for respiration is essential only when ethanol or glycerol is the sole carbon source. Here, we have grouped the essential genes into four categories, depending on the circumstances or conditions these genes are required (Fig. 1).

**(1) Conditional essential:** these genes are essential only under certain circumstances or growth conditions. Auxotrophic genes are among the best examples for conditional essential. In the absence of uracil in culture medium, the orotidine-5'-phosphate decarboxylase gene, *URA3*, which catalyzes the sixth enzymatic step in the *de novo* biosynthesis of pyrimidines, becomes essential 3. Many genes could be conditionally essential, but the “condition” is unknown (see below for discussion about *PKC1*). This could be one of the reasons why the molecular functions of more than 10% of the yeast protein-coding genes are still unknown. Many of these genes could be required or essential only under certain conditions, or stresses.

**(2) Essential:** refers to genes required for survival under the optimal growth condition. This is the most widely accepted definition of essential genes. The “optimal condition” though, is somewhat debatable. The yeast *PKC1* gene, for example, encodes a protein serine/threonine kinase, which is essential for cell wall remodeling during growth 4. Deletion of *PKC1* is lethal, but the lethality could be recovered by an osmotic stabilizer (such as sorbitol) 5, the “optimal growth condition” for this mutant. Another example is the Acetyl-CoA synthetase gene *ACS2*, which is required for fermentative growth on glucose. Null mutant is inviable in YPD medium, but is capable of respiratory growth on non-fermentable carbon sources, such as ethanol 6. Therefore, the “optimal growth condition” for *acs2*Δ, is non-fermentable carbon sources, rather than glucose.

The percentage of genes that are essential in the whole genome varies in different species, ranging from ~2% in *Drosophila melanogaster* 7 to about 80% in *Mycoplasma genitalium* 8. In the yeast *Saccharomyces cerevisiae*, about 20% of the whole genes are essential. The extremely low percentage of essential genes in *Drosophila* could be attributed to the methods (RNAi) used. RNAi is far less restrictive than the knock-outs used in *S. cerevisiae* and sometimes leads to hypomorphic results only 7. Because of the importance of essential genes, yeast may have even evolved a protection mechanism to reduce the mutation rate of essential genes by placing them in the cold spots for mutation 9.

Most of the gene products, essential or not, form protein complexes to carry out their molecular functions 10. A recent study showed that essential protein complexes tend to be formed by essential genes and nonessential complexes by nonessential genes, suggesting the existence of modular essentiality 11.

There are also cases where an essential gene becomes non-essential by deletion of another (non-essential) gene. *FtsZ* is a tubulin-like gene in bacteria that forms the cytoskeletal framework of the cytokinetic ring. It is essential for cell division in wild type bacteria. However, in the L-form of *Bacillus subtilis*, caused by a single amino acid mutation of *yqiG* gene, *FtsZ* becomes non-essential. The *B. subtilis* cells proliferate by extrusion and resolution, instead of binary fission 12.

**(3) Redundant essential (synthetic lethal/sick):** eukaryotic cells evolve multiple redundant pathways to resist environmental and genetic perturbations 13. One way of producing a redundant pathway is the so called whole genome duplication, a product of nondisjunction during meiosis, which results in additional copies of the entire genome 14. Gene duplication may also provide stability to an organism by buffering the effect of harmful mutations 15. The *Saccharomyces* genome contains ~1,060, or ~530 pairs of paralogs, homologous sequences evolved from gene duplication. Synthetic lethality occurs when mutations of both genes cause cell death, while single deletion of either gene is viable. The paralogous genes *BDF1* and *BDF2*, for example, encode bromodomain-containing transcription factors 16. Double deletion is lethal, while single deletion of either *BDF1* or *BDF2* has no apparent phenotype 17. In addition, *bdf1*Δ is sensitive to salt stress and the sensitivity can be suppressed by over-expression of *BDF2* 18. Not all paralogous genes, however, are synthetic lethal. The paralogs *CYC1* and *CYC7* encode two isoforms of cytochrome c, iso-1-cytochrome c and iso-2-cytochrome c, respectively. Iso-1-cytochrome c and iso-2-cytochrome c constitute approximately 95% and 5% of the total amount of cytochrome c in aerobically grown cells 19. Double deletion of *CYC1* and *CYC7* completely blocks growth on non-fermentable carbon sources, but the growth is normal on rich glucose medium 20. As for essentiality, *BDF1/BDF2* should be considered as redundant essential, while *CYC1/CYC7* should be considered as conditional essential. Another example of conditional essential is *PET9*, which encodes the major ADP/ATP translocator of the mitochondrial inner membrane 21. *PET9* has two paralogs, *AAC1* and *AAC3*, both are expressed at a lower level compared to *PET9*. Similar to *CYC1/CYC7* double deletion, the triple deletion of *PET9/AAC1/AAC3* is inviable only under anaerobic conditions 22. A study in *Caenorhabditis elegans* showed that the non-essential (conditional essential) genes are actually duplicated more often, while essential genes (become redundant essential after duplication) are less often duplicated 15. It is unclear how many of the 530 pairs of paralogs in yeast were evolved from non-essential (conditional essential), or essential genes.

**(4) Absolute essential (minimal genes):** genes that are necessary and sufficient for supporting cellular life in an environment that provides all necessary nutrients and is free from any stress 23. These genes are often referred as minimal genes, and collectively as the minimal gene set. The “stress-free” environment, although easily defined, is difficult to implement in practice, since it is close to impossible to create such a “stress-free” environment.

The parasitic bacterium *Mycoplasma genitalium* has an extremely small genome of ~580 kb, containing only 482 protein-coding genes. It offers one of the best organisms to study minimal genes 24. Among the 482 genes, 382 were identified as essential and 256 were suggested as minimal genes 25. These minimal genes are mainly involved in DNA replication, protein translation and metabolism. A set of genes required for recombination and DNA repair was also identified in the minimal genes 25, suggesting that DNA repair is a fundamental process required for cell survival.

## THE ESSENTIAL FUNCTIONS OF THE ESSENTIAL GENES

To survive, an organism must be able to perform at least two fundamental functions: obtain energy, and reproduce. Cells must convert nutrients from foods, such as proteins, fats and sugars, into adenosine triphosphate (ATP), the ultimate source of energy for living cells. This process of energy conversion is referred as metabolism. Gene ontology (GO) analysis 26 of the 1120 essential genes of *Saccharomyces cerevisiae* 27 indicates that about 74 % of the essential genes are involved in metabolic process and at least 14% in cell cycle regulation, the two essential functions for cell survival. To keep its simplicity, the discussion below only refers to these essential genes, unless noted otherwise. It should be noted that the genome-wide deletion set for *Saccharomyces* 27 also contains multiple mutations that can affect phenotypes.

## Essential genes and metabolism

More than 700 essential genes are involved in this process that is essential for converting nutrients and providing energy for cells. It includes both catabolism (about 120 genes) and biosynthesis (about 450 genes) of nucleic acids, proteins, carbohydrate, lipids and other macromolecules. Among the ~120 catabolic related genes, 17 are involved in autophagy, a process for macromolecule recycling and intracellular structure remodeling. Recent studies have shown that autophagy plays a much greater physiological role, such as response to starvation and other stress conditions 28,29. In addition to the 17 essential genes, there are about 70 non-essential genes (including conditional essential and redundant essential, such as *ATG33* and *SCM4*) involved in autophagy.

## Essential genes and cell division/reproduction

Reproduction is one of the fundamental functions for cell survival. Among the 158 (14%) cell cycle related essential genes, the most important processes of these genes involved include: 1) Mitotic cell cycle, 2) Cytoskeleton organization, and 3) Response to stress and stimulus.

*1) Mitotic cell cycle:* Not surprisingly, more than half of the essential genes in mitosis are involved in nuclear division, including DNA replication, DNA repair, spindle assembly checkpoint, chromosome condensation and chromosome segregation. Yeast can reproduce either via asexual reproduction (mitosis) or sexual reproduction (meiosis). Meiosis, therefore, is not an essential function for cells to survive. It was puzzling to see 39 out of the 158 cell cycle-related essential genes regulate meiosis. Further analysis revealed that none of the 39 genes are meiotic specific. They are involved in both mitosis and meiosis. The cohesin maintenance factor gene *PDS5*, for example, is required for sister chromatid condensation and cohesion during mitosis 30,31. It is also required for sister chromatid cohesion in meiosis 32. Some genes, such as *DSN1*, which encodes an essential component of the MIND kinetochore complex 33, are essential for meiosis 34, but their essentiality for survival is more likely due to their functions in mitosis.

*2) Cytoskeleton organization:* *Saccharomyces cerevisiae* reproduces through budding, a stereotypical pattern of polarized growth. Cytoskeleton organization plays an essential role in all steps of cell growth and cell division. The microtubule cytoskeleton related proteins (such as *IPL1*, and *SPC105*) are mostly required for nuclear division, including spindle checkpoints. The actin cytoskeleton related proteins are involved in organelle localization, vesicle transport and cytokinesis, such as the septin ring organization. The actin cytoskeleton of budding yeast consists of both the actin cortical patch and the actin cables. The actin cortical patches show a polarized distribution while the actin cables are oriented along the mother-bud axis during cell division 35.

*3) Response to stress or stimulus:* Faithful DNA replication and transmission of hereditary information is critical to cell survival. Not surprisingly, the majority of these essential stress-response genes are involved in genome integrity checkpoints, DNA damage and DNA repair. Rad53, for example, is a DNA damage response protein. It is required for cell cycle checkpoint function in response to DNA damage 36.

## THE “NON-ESSENTIAL” FUNCTIONS OF THE ESSENTIAL GENES

### Essential genes and cell death

Cell death, as opposed to cell survival, is not an essential function. GO analysis, however, revealed that at least five essential genes, *BIR1*, *MCD1*, *ESP1*, *PDS5* and *CDC48* are involved in cell death in budding yeast. Cautions should be taken when using the GO analysis. While it works well on large scales, it may provide inaccurate information on specific genes. *PET9* for example, is listed as one of the essential genes involved in cell death. *PET9* is required for mitochondrial outer membrane permeabilization and cytochrome c release 37, it is a conditional essential, rather than an essential gene.

Interestingly, all five cell death-related genes play critical roles in mitotic nuclear division. Induction of apoptotic cell death is likely a result of their “secondary” functions or simply by loss of function. Bir1, for example, is a homolog of human survivin and a subunit of the chromosomal passenger complex, which regulates chromosome segregation 38,39. Under oxidative stress, however, the yeast Bir1 becomes a substrate for Nma111, the homologue of the human pro-apoptotic serine protease Omi/HtrA2BIR1, which mediates apoptotic cell death 40. Deletion of *BIR1* also causes malfunction of the spindle assembly checkpoint and apoptosis 41, suggesting a possible connection between the functions in checkpoint regulation and anti-apoptosis.

**Figure 2:**
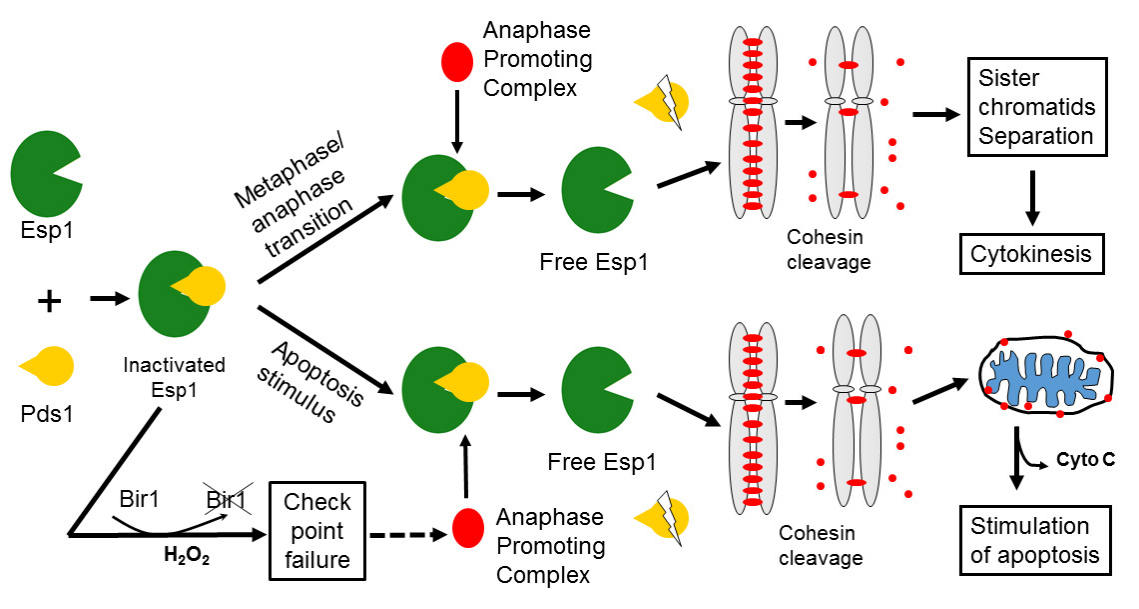
FIGURE 2: Involvement of essential genes (ESP1, MCD1 and BIR1) in apoptotic cell death. Modified according to 43.

The yeast Esp1 is a caspase-like cysteine protease 42. During metaphase-anaphase transition, Esp1 is released by its inhibitor Pds1, triggering sister chromatid separation by cleavage of the cohesin protein Mcd1. Upon oxidative stress, the Esp1 is prematurely released and cleaves Mcd1. The C-terminal fragment of Mcd1 is translocated into mitochondria and triggers the release of cytochrome c and consequently apoptotic cell death (Fig. 2) 43, suggesting that Mcd1 actively participates in cell death. It would be interesting to see if any other essential proteins perform similar functions, i.e., triggering cell death under stress conditions, to ensure their essentiality.

**Figure 3:**
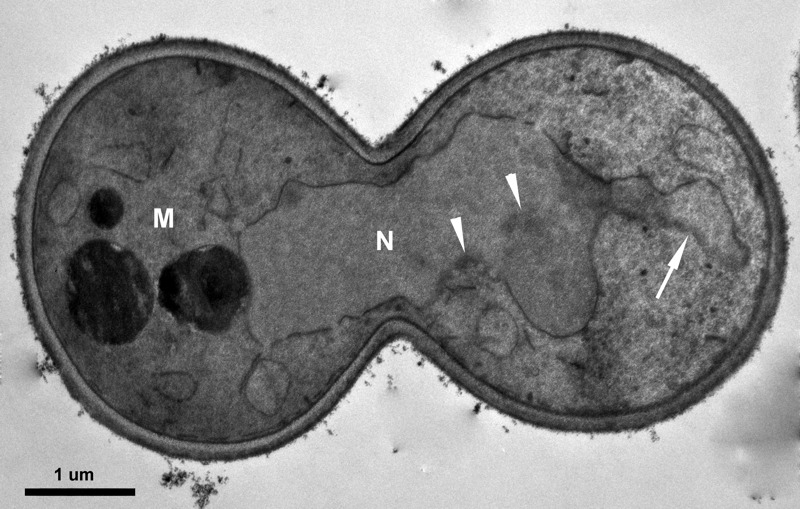
FIGURE 3: TEM image of pds5-1 mutant showing the nucleus unable to divide when switched to non-permissive temperature. It also shows chromatin condensation (arrowhead) and nucleus with protruding tube (arrow), both typical markers of apoptotic cell death. N = nucleus. M = mother cell, as suggested by the vacuole.

Because of the nature of the essential genes - cells die without them, a still unanswered question is how cells die in the absence of an essential gene. Studies indicate that mutants of yeast essential genes could die apoptotically. In fact, the very first paper published on yeast apoptosis by Frank Madeo and his colleagues involved *CDC48*, an essential gene that regulates mitotic spindle disassembly. In this pioneer study, they reported that mutants in *CDC48* show typical markers of apoptosis 44. Mutation of another essential gene, *PDS5*, was also reported to lead to features of apoptosis (Fig. 3) 45,46. What is unclear is whether the apoptotic cell death caused by mutation of *CDC48* or *PDS5* is due to the loss of functions of the essential gene, or like *MCD1*, the essential gene actively participates in the cell death. If it is due to the loss of function, what are the triggers or inducers of the cell death?

In addition to cell death, GO analysis revealed that five essential genes are related to aging, another “non-essential” function for cell survival. Similar to genes in cell death, all these five essential genes (*ACT1*, *DNA2*, *NMT1*, *CDC25* and *PGA3*) are involved in other essential functions and defects could lead to accumulation of damage that leads to aging. *ACT1* for example, encodes the essential gene for actin, a cytoskeleton protein critical for many cellular processes, such as polarized cell growth 47. It has been shown that enhanced actin dynamics can increase the yeast lifespan, while decrease of actin dynamics causes depolarization of the mitochondrial membrane, production of reactive oxygen species (ROS) and cell death 48. Mutation of *CDC25*, on the other hand, enhances the lifespan in both yeast 49 and mice 50. *CDC25* encodes a guanine nucleotide exchange factor, which is required for glucose metabolism by activing the Ras GTPases 51. Mutation of *CDC25* reduces glucose/calorie consumption and possibly reduces the level of ROS 49.

### Essential genes and mitochondrial organization

Mitochondria are organelles present in virtually all eukaryotic cells. They are the site for numerous important cellular metabolic processes. Although *S. cerevisiae* is able to carry out fermentative growth on carbon sources such as glucose in the absence of mitochondrial DNA, maintenance of the mitochondrial compartment is essential for survival. GO analysis revealed that about 50 essential genes are involved in mitochondrial organization. A systematic large screening of the 768 essential genes with a regulatable promoter identified about 120 genes that are essential for mitochondrial morphology 52. The majority of these genes are involved in protein targeting/import/transport to mitochondria. Most prominently are the TOM (translocase of outer membrane) proteins (TOM20, TOM22 and TOM40), and the TIM (translocase of inner membrane) proteins (TIM10, TIM12, TIM17, TIM22, TIM23, TIM44, TIM50 and TIM54). These are essential proteins of either the outer or inner mitochondrial membrane that are important for import/insertion of proteins into the mitochondrial inner membrane and to the mitochondrial matrix.

Besides producing ATP as the energy source, mitochondria are involved in a number of essential biosynthetic pathways, such as the ergosterol biosynthetic pathway (encoded by *ERG29*), which is essential to yeast viability 53. Mitochondria also play a central function in the biogenesis of cellular iron-sulphur proteins, such as Rli1, which is required for ribosome biogenesis and protein translation 54. Recent studies also demonstrated that mitochondria are key regulators of apoptotic cell death 55,56,57.

## CONCLUSIONS AND PERSPECTIVES

The reason essential genes are essential is that these genes carry out biological functions that are necessary for cell survival. Many of the “non-essential genes” also perform essential functions. They are deemed non-essential, not because of their functions, but rather their functions are backed up with redundant genes or pathways. Therefore, the definition of essential genes should be expanded to include all genes that carry out essential functions, rather than simply required for survival. The difficulty of using this term is that a lot of essential biological functions are yet to be uncovered, a challenge and a future direction towards a better understanding of cell biology. Another challenge is to decipher the essential function(s) each gene carries. The problem for the “non-essential”, or conditionally essential genes, is to identify the unknown conditions/circumstances under which the genes are required, because mutants deleted for these genes lack any obvious phenotype under normal conditions. The challenge for essential genes is that death is the only “phenotype” in the absence of any essential gene. The generation of the tetracycline-regulatable alleles of essential genes 58 greatly facilitates large scale studies on essential genes, such as those involved in secretion 59, mitochondria 52 or G-protein signaling 60. These studies often identify the non-essential, or secondary functions of the essential genes, which may help further identify the essential functions. The essential gene *YPP1/YGR198W* was first discovered as a gene required for protection from α-synuclein, a protein that causes Parkinson’s disease in human 61. It is also shown that *YPP1* interacts with genes involved in protein sorting and secretion, which further led the discovery of its primary essential functions 62,63. In addition to discovery of the essential functions, it is important to understand their involvement in cell death. Do all the essential genes actively participate in cell death? Is there a universal trigger of cell death in the absence of an essential gene?
